# Association between vitamin D receptor gene polymorphisms and susceptibility to tuberculosis: a systematic review and meta-analysis

**DOI:** 10.3389/fgene.2024.1382957

**Published:** 2024-08-20

**Authors:** Rongshan Tao, Shujuan Xiao, Lianping Wang, Chunjie Hu, Huiqin Suo, Ruiyu Long, Hangyu Liu, Wei Luo, Feng Hong, Jingming Zhao, Qingjie Li

**Affiliations:** ^1^ The Key Laboratory of Environmental Pollution Monitoring and Disease Control, School of Public Health, Ministry of Education, Guizhou Medical University, Guiyang, China; ^2^ Xiangya School of Public Health, Central South University, Changsha, China; ^3^ School of Traditional Chinese Medicine Jilin Agricultural Science and Technology University, Changchun, China; ^4^ School of Life Sciences, Jilin University, Jilin, China; ^5^ Anorectal Center, The Affiliated Hospital to Changchun University of Chinese Medicine, Changchun, China; ^6^ School of Pharmacy, Changchun University of Traditional Chinese Medicine, Changchun, China; ^7^ Office of Infection Control, The Affiliated Hospital to Changchun University of Chinese Medicine, Changchun, China; ^8^ Proctology Department, Affiliated Hospital of Changchun University of Chinese Medicine, Changchun, China; ^9^ Research Center of Traditional Chinese Medicine, The Affiliated Hospital to Changchun University of Chinese Medicine, Changchun, China

**Keywords:** tuberculosis, gene polymorphisms, VDR, vitamin D receptor, meta-analysis

## Abstract

**Objective:**

Tuberculosis (TB) is the leading cause of mortality worldwide. Previous studies have reported that TB susceptibility can be caused by vitamin D deficiency, which is affected by polymorphisms in the vitamin D receptor (*VDR*) gene. However, these results have been inconsistent. Therefore, we performed a meta-analysis to investigate the association between *VDR* polymorphisms and TB susceptibility.

**Methods:**

We systematically searched for relevant literature in PubMed, Embase, and Medline databases through December 31st, 2022. Inclusion and exclusion criteria were made to ensure that HIV-negative population is the targeted subjects. The pooled odds ratio (*OR*) and 95% confidence interval (*CI*) were then used to assess the strength of the association, and the quality of the included articles was evaluated using the Newcastle–Ottawa Scale. Potential sources of heterogeneity were evaluated based on subgroup and meta-regression analyses.

**Results:**

In our meta-analysis, we found that the FokI polymorphism in the *VDR* gene was associated with increased TB susceptibility in the allele and recessive genotype models (*OR* f vs. F = 1.235, 95%*CI*: 1.035–1.475; *OR* ff vs. Ff + FF = 1.317, 95%*CI*: 1.005–1.727. Further subgroup analysis based on ethnicity demonstrated the association with the risk of TB in all genotype models of the FokI polymorphism for Han population. Meta-regression analysis also indicated that ethnicity could be a potential source of heterogeneity in the FokI and BsmI polymorphisms in the *VDR* gene. However, publication year was another source of heterogeneity for the *Taq*I polymorphism.

**Conclusion:**

In summary, the *Fok*I polymorphism in the *VDR* gene was found to increase the risk of TB in the HIV-negative population, both overall and in Asian populations. The findings presented in this paper could provide clues for preventing TB from the perspective of vitamin D supplementation, which is a controversial topic in the field of medicine and health.

## Introduction

Tuberculosis (TB) is a communicable disease caused by the *Mycobacterium tuberculosis* complex (MTB). It is considered a major determinant of poor health and one of the leading causes of mortality, responsible for 1.6 million deaths worldwide in 2021 (Möller and Hoal, 2010). According to “*Global Tuberculosis Report 2022*” from the World Health Organization, approximately 10.6 million people worldwide were infected with TB in 2021, representing a 4.5% increase from the 10.1 million cases recorded in 2020. Similarly, the report cited an estimated 3.6% increase in TB incidence, to approximately 134 cases per 100,000 population, between 2021 and 2020 (WORLD HEALTH ORGANIZATION, 2022-10). These challenges highlight the serious issue associated with preventing and controlling TB epidemics.

The persisting association between MTB and its host implies that this pathogen has evolved extensive mechanisms to evade elimination by the immune system. Accordingly, it causes no substantial harm and is not transmitted until immune system responses decline due to co-infections or other factors. As a result, despite approximately one-quarter of the global population being infected with MTB, only 5%–10% of these individuals develop TB (Delgado et al., 2002; Dye et al., 1999). Moreover, the process of TB might also be associated with other factors, such as lifestyle, environment, and genetics (Hillerdal, 2000; Newport and Nejentsev, 2004; Nava-Aguilera et al., 2009). Among these, genetic factors of the host play a vital role in susceptibility or resistance to TB.

In recent decades, vitamin D has been shown to play an essential role in bone health (Ganmaa et al., 2020). Moreover, recent studies based on different populations have indicated that vitamin D deficiency increases the risk of developing TB. Vitamin D also plays a role in the biological modulation of the immune system in response to TB. Here, 1,25-dihydroxyvitamin D_3_ (the active form of vitamin D) is activated by 1 α -hydroxylase, which can be expressed by macrophages and other immune cells. Further, evidence suggests that the cellular functions of 1,25-dihydroxyvitamin D_3_ can be affected by variations in the vitamin D receptor (*VDR*) gene (L Bishop et al., 2020).

**FIGURE 1:**
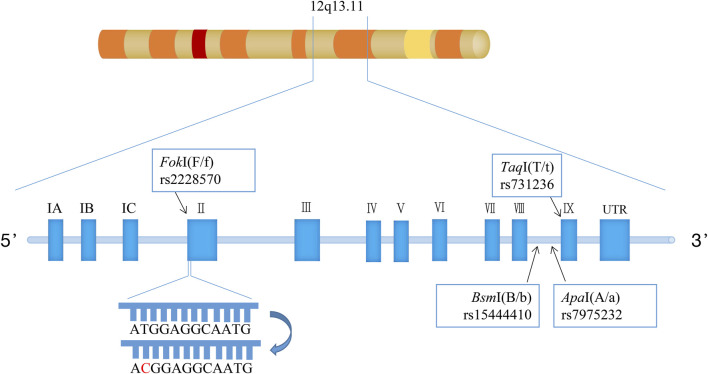
Genomic region and exon-intron structure of the vitamin D receptor (VDR) gene. (The VDR gene is placed on human chromosome 12q13.11, contains nine exons and encompasses various single nucleotide polymorphisms (SNPs) including *Fok*I (F/f), *Bsm*I (B/b), *Apa*I (A/a), *Taq*I (T/t). The presence of a T/C transition polymorphism (ATG to ACG) at the first of two potential translation initiation sites in exon II).

*VDR* is located on the long arm of chromosome 12q13 (Miyamoto et al., 1997). Polymorphisms of this gene are observed across various population groups, although the prevalence of specific *VDR* genotypes varies among populations. Several polymorphisms, including *Bsm*I (rs1544410), *Apa*I (rs7975232), and *Taq*I (rs731236), at the 3′end of *VDR* with strong linkage disequilibrium have been examined. Despite having no impact on the structure of the expressed VDR protein, these three single nucleotide polymorphisms potentially have a role in regulating the expression of the *VDR* gene. Another gene polymorphism, *Fok*I (rs2228570), is located in exon 2, at a translation initiation site, and is anticipated to alter the structure of the encoded protein (Figure 1) (Shaikh et al., 2016).

Multiple studies have investigated the potential effect of *VDR* gene polymorphisms on susceptibility to TB; however, the results of these studies have been inconsistent. This inconsistency could be due to various factors, such as small sample sizes, insufficient power to detect associations between *VDR* gene polymorphisms and susceptibility to TB, the study design, the ethnicity of the study population, and the genetic context. A meta-analysis, a statistical technique that combines multiple results from previous studies to increase the statistical power and improve the precision of the estimation of pooled data (Blettner et al., 1999), could thus be a good option for analyzing inconsistent results.

Several meta-analyses have been conducted to identify the potential association between *VDR* gene polymorphisms and TB susceptibility over the past decades; however, larger pooled datasets are required to improve the power of effect estimates. Furthermore, few studies have been performed to uncover the impact of *VDR* gene polymorphisms based on different ethnic backgrounds. Therefore, we performed a comprehensive meta-analysis to (1) systematically evaluate the relationship between *VDR* gene polymorphisms, including *Fok*I, *Bsm*I, *Apa*I, *Taq*I, and TB susceptibility, (2) explore the potential effect of *VDR* gene polymorphisms on TB susceptibility in various ethnic groups.

## Materials and methods

### Study selection

The PubMed, Embase, and MEDLINE databases were searched for studies to include in this meta-analysis. The keyword used were: “VDR”, “Vitamin D receptor”, “tuberculosis”, “gene”, and “polymorphism.” The reference lists of the review articles were also manually searched for additional pertinent publications. The article search was conducted for articles published until 30 December 2022.

### Inclusion and exclusion criteria

The literature was included based on the following criteria: i) case-control studies assessing the association between *VDR* gene polymorphisms and TB risk; ii) all participants in the studies confirmed to be negative for human immunodeficiency virus (HIV-negative), which could be examined in accordance with a certain diagnostic criterion of laboratory or antibody tests; iii) sufficient data on alleles and genotypes for the case and control groups provided to calculate the odds ratios (*OR*s) and 95% confidence intervals (*CI*s). The exclusion criteria were as follows: i) studies of control groups with gene distributions that deviated from the Hardy–Weinberg equilibrium (HWE) (Mayo, 2008); ii) low-quality studies (i.e., Newcastle–Ottawa Scale (NOS) scores (Stang, 2010) below 6); iii) review articles, abstracts, animal experiments, letters, editorials, case reports, and non-English publications.

### Data extraction and quality assessment

According to the predetermined data extraction sheet, the following data were extracted independently by two researchers (R.S. Tao and S.J. Xiao): first authors’ names, year of publication, country of origin, ethnicity, the total number of participants in the case and control groups, genotype and allele frequencies in the case and/or control groups, mean or range of age, genotyping method, and TB type. In case of discrepancies, a third reviewer (L.P. Wang) concluded on the extracted data. For quality assessments, the NOS was used. Studies were stratified into two categories, specifically low quality (scores 0–5) and high quality (scores ≥6).

### Statistical analysis

A chi-square test was used to assess the deviation from the HWE in terms of allele and genotype frequencies in the control groups. The strength of the association between *VDR* polymorphisms and TB susceptibility was evaluated by calculating the pooled OR and its 95% CI. Data were extracted to build different comparison genotype models for the polymorphisms (i.e., *Fok*I, *Bsm*I, *Apa*I, *Taq*I) of the *VDR* gene, as follows: i) *Fok*I, allele model (f vs. F), dominant model (ff + Ff vs. FF), recessive model (ff vs. Ff + FF), homozygote model (ff vs. FF); ii) *Bsm*I, allele model (b vs. B), dominant model (bb + Bb vs. BB), recessive model (bb vs. Bb + BB), homozygote model (bb vs. BB); iii) *Apa*I, allele model (a vs. A), dominant model (aa + Aa vs. AA), recessive model (aa vs. Aa + AA), homozygote model (aa vs. AA); iv) *Taq*I, allele model (t vs. T), dominant model (tt + Tt vs. TT), recessive model (tt vs. Tt + TT), homozygote model (tt vs. TT). Heterogeneity among studies was measured based on the *Q* statistic (a *p*-value with a significance level of 0.05) and the *I* ^*2*^ statistic, which was used to quantify the inconsistency between study results. Commonly, a fixed-effects model for the pooled *OR* is used for a *Q* statistic with *p* > 0.05 and *I* ^*2*^ < 50%. Otherwise, a random-effects model is used to combine the data if *p* ≤ 0.05 and *I* ^*2*^ ≥ 50%. Subgroup analysis was performed to evaluate the source of heterogeneity from the perspective of ethnicity, and meta-regression analysis was performed to explore the potential sources of heterogeneity based on the publication year and ethnicity. The stability of our results was assessed using a sensitivity analysis, and potential publication bias was evaluated using funnel plots and Egger’s test (Egger et al., 1997). The data for this study were analyzed using R language programming software (version 4.2.3).

## Results

### Characteristics of the eligible studies

**FIGURE 2:**
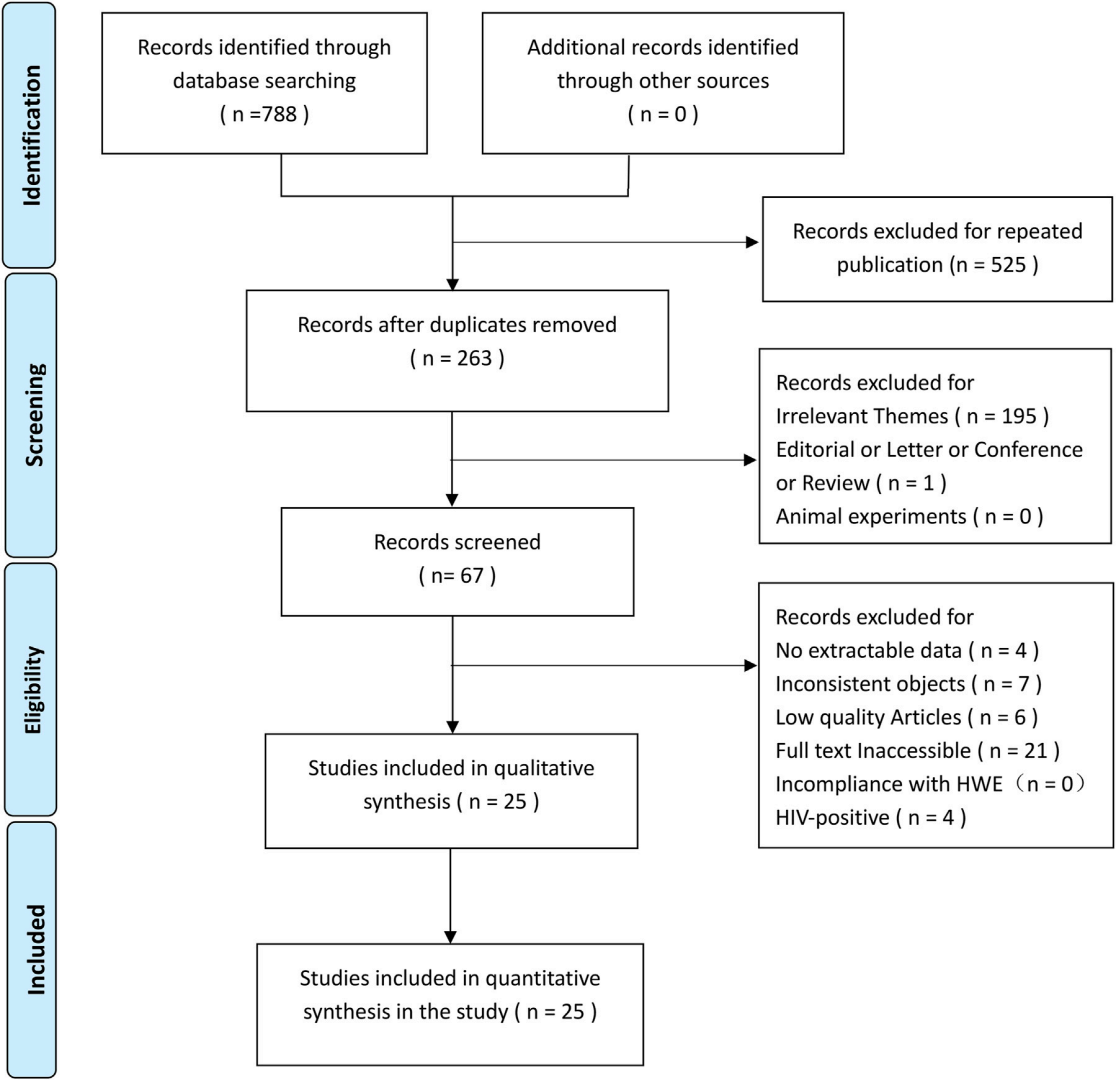
PRISMA flflow chart.

In total, 788 articles were identified via a systematic literature search of the PubMed, Embase, and MEDLINE databases. After screening using our inclusion and exclusion criteria, 25 eligible articles (Delgado et al., 2002; Liu et al., 2004; Roth et al., 2004; Merza et al., 2009; Banoei et al., 2010; Marashian et al., 2010; Zhang et al., 2010; Ates et al., 2011; Kang et al., 2011; Wu et al., 2013b; Joshi et al., 2014; Sinaga et al., 2014; Fernández-Mestre et al., 2015; Salimi et al., 2015; Jafari et al., 2016; Lee et al., 2016; Rong et al., 2017; Wang et al., 2017; Devi et al., 2018; Zhang et al., 2018; Panda et al., 2019; Silva-Ramírez et al., 2019; Hidayah et al., 2021; Varzari et al., 2021; Wani et al., 2021) were included (Figure 2: PRISMA flow diagram). Of these, 20 studies (Delgado et al., 2002; Liu et al., 2004; Merza et al., 2009; Banoei et al., 2010; Marashian et al., 2010; Zhang et al., 2010; Kang et al., 2011; Wu et al., 2013a; Joshi et al., 2014; Sinaga et al., 2014; Fernández-Mestre et al., 2015; Salimi et al., 2015; Lee et al., 2016; Rong et al., 2017; Wang et al., 2017; Devi et al., 2018; Zhang et al., 2018; Panda et al., 2019; Hidayah et al., 2021; Wani et al., 2021) were from Asia and the remaining five were from Europe (Varzari et al., 2021; Ates et al., 2011), North America (Silva-Ramírez et al., 2019), and South America (Jafari et al., 2016; Roth et al., 2004). The case and control groups consisted of 3,768 and 3,742 patients, respectively. Among these articles, 22 and eight studies provided information on the FokI and ApaI polymorphisms, respectively, and 15 studies provided information on the BsmI and TaqI polymorphisms. The NOS scores of the included studies ranged from 6 to 9. Tables 1, 2 summarize the basic characteristics of genotype and allele frequencies in the included studies, respectively.

**TABLE 1:** Characteristics of individual studies included in the meta-analysis.

Author (year)	Country	Ethnicity	Case/Control	Age^a^	Type of TB^b^	Genotyping	Polymorphism(s)^c^	NOS^d^ (score)
Hidayah 2021	Indonesia	Asian	83/118	41.0/NA	PTB	PCR	1,2,3,4	8
Varzari 2021	Moldova	European	272/251	40.7/47.6	PTB	PCR-RFLP	1,3,4	6
Wani 2021	India	Asian	100/102	45.9/NA	EPTB	PCR-RFLP	2	6
Panda 2019	India	Asian	150/150	39.4/32.1	PTB	PCR-RFLP	1	7
Devi 2018	India	Asian	169/227	32.6/33.4	PTB	PCR-RFLP	1,2,3,4	6
Silva 2019	Mexico	North American	257/457	45.3/36.5	PTB	TaqMan	1,2,3,4	7
Zhang 2018	China	Asian	108/59	38.0/49.3	TB	PCR-RFLP	1,2,3,4	7
Rong 2017	China	Asian	258/291	56.3/29.6	TB	iMLDR	3	6
Wang 2017	China	Asian	150/149	46.3/45.8	STB	PCR-RFLP	1	6
Jafari 2016	Iran	Asian	96/122	51.0/48.0	PTB	ARMS-PCR	1,2,3,4	7
Lee 2016	Taiwan	Asian	198/170	55.8/55.8	PTB	TaqMan	1,2,3,4	7
Fernandez 2015	Venezuela	South American	93/102	17-70/20-67	PTB	PCR-RFLP	1,2,4	7
Salimi 2015	Iran	Asian	120/131	51.5/48.1	PTB	PCR-RFLP	1,2,3	9
Sinaga 2014	Indonesia	Asian	76/76	NA	PTB	PCR-RFLP	1,3	9
Joshi 2014	India	Asian	110/115	25.0/21.6	PTB	PCR-RFLP	1,3	7
Wu 2013	China	Asian	213/211	18-72/NA	PTB	PCR-RFLP	1,2	8
Ates 2011	Turkey	European	128/80	47.8/54.1	PTB, EPTB	PCR-RFLP	1,2,3	6
Kang 2011	South Korea	Asian	155/105	17-69/21-52	PTB	PCR-RFLP	1,2,3	6
Banoei2010	Iran	Asian	60/62	45.8/41.0	PTB	PCR	1,2,3	6
Marashian2010	Iran	Asian	164/50	NA	TB	PCR-RFLP	1	6
Zhang 2010	China	Asian	110/102	33.8/32.2	STB	PCR-RFLP	1	9
Merza 2009	Iran	Asian	117/60	NA	PTB	PCR-RFLP	1,3	6
Roth 2004	Peru	South American	103/206	25.4/25.4	PTB	PCR-RFLP	1,2	7
Liu 2003	China	Asian	120/240	27.7/27.3	PTB	PCR-RFLP	1	9
Delgado 2002	Cambodia	Asian	358/106	42.2/37.5	PTB	PCR-RFLP	2	6

“NA” means that the data were not available.

^a^ Age was shown as the mean or range age of cases and controls.

^b^ PTB, pulmonary tuberculosis; EPTB, extrapulmonary tuberculosis; STB, spinal tuberculosis.

^c^ 1:*FokI*; 2:*TaqI*; 3:*BsmI*; 4:*ApaI*.

^d^ Newcastle-Ottawa scale.

**TABLE 2:** Distributions of VDR genotype and allele among TB patients and controls.

A VDR *Fok*I (rs2228570)											
First author reference	Case			Control			Case		Control		
	FF	Ff	Ff	FF	Ff	Ff	F	f	F	f	HWE
Ates	58	60	10	35	37	8	176	80	107	53	0.926
Devi	59	106	4	119	90	18	224	114	328	126	0.986
Hidayah	20	46	17	46	62	10	86	80	154	82	0.412
Joshi	51	46	13	63	41	11	148	72	167	63	0.539
Lee	44	104	50	51	87	32	192	204	189	151	0.893
Panda	55	58	37	86	51	13	168	132	223	77	0.410
Salimi	65	44	11	93	31	7	174	66	217	45	0.157
Silva	76	119	62	80	218	159	271	243	378	536	0.939
Sinaga	27	42	7	30	34	12	96	56	94	58	0.210
Wang	22	53	75	39	68	42	97	203	146	152	0.570
Liu	29	63	28	85	120	35	121	119	290	190	0.781
Zhang	16	43	51	26	47	29	75	145	99	105	0.576
Fernandez	34	47	12	26	60	16	115	71	112	92	0.165
Jafari	41	50	5	55	61	6	132	60	171	73	0.105
Wu	72	96	45	101	88	22	240	186	290	132	0.910
Varzari	100	117	47	74	125	44	317	211	273	213	0.784
Zhang	21	80	79	21	25	13	122	238	67	51	0.735
Banoei	30	21	9	29	27	6	81	39	85	39	0.997
Kang	30	58	15	41	43	21	118	88	125	85	0.306
Marashain	97	57	10	15	30	5	251	77	60	40	0.210
Merza	67	46	4	35	25	0	180	54	95	25	0.125
Roth	9	32	59	14	78	109	50	150	106	296	0.923

B VDR *Bsm*I (rs1544410)											
First author reference	Case			Control			Case		Control		
	BB	Bb	bb	BB	Bb	bb	B	b	B	b	HWE
Ates	28	68	32	5	38	37	124	132	48	112	0.504
Devi	24	100	45	56	113	58	148	190	225	229	0.998
Hidayah	63	18	2	83	35	0	144	22	201	35	0.317
Lee	183	14	1	146	24	0	380	16	316	24	0.612
Salimi	31	66	23	39	70	22	128	112	148	114	0.609
Silva	153	96	8	273	168	16	402	112	714	200	0.274
Sinaga	0	52	24	2	18	56	52	100	22	130	0.140
Rong	137	101	20	188	86	17	375	141	462	120	0.253
Jafari	11	42	43	15	52	55	64	128	82	162	0.884
Varzari	108	121	40	94	119	34	337	201	307	187	0.931
Zhang	2	19	159	1	4	54	23	337	6	112	0.073
Banoei	13	27	20	31	26	5	53	67	88	36	0.990
Kang	2	13	135	0	8	75	17	283	8	158	0.899
Merza	7	67	43	13	21	26	81	153	47	73	0.121

C VDR *Taq*I (rs731236)											
First author reference	Case			Control			Case		Control		
	TT	Tt	tt	TT	Tt	tt	T	t	T	t	HWE
Ates	49	65	14	30	39	11	163	93	99	61	0.957
Devi	86	73	10	116	86	25	245	93	318	136	0.342
Hidayah	72	11	0	97	19	2	155	11	213	23	0.831
Lee	186	12	0	149	20	1	384	12	318	22	0.935
Salimi	52	54	14	67	50	14	158	82	184	78	0.607
Silva	132	110	15	228	199	30	374	140	655	259	0.304
Wani	54	42	4	60	33	9	150	50	153	51	0.383
Delgado	325	30	3	96	10	0	680	36	202	10	0.878
Fernandez	51	33	2	58	38	1	135	37	154	40	0.153
Jafari	38	46	12	56	58	8	122	70	170	74	0.386
Zhang	160	19	1	52	7	0	339	21	111	7	0.889
Kang	134	14	1	85	8	1	282	16	178	10	0.323
Banoei	8	33	19	33	24	5	49	71	90	34	0.977
Roth	90	10	0	169	31	1	190	10	369	33	0.992

D VDR *Apa*I (rs7975232)											
First author reference	Case			Control			Case		Control		
	AA	Aa	aa	AA	Aa	aa	A	a	A	a	HWE
Devi	36	83	50	49	103	75	155	183	201	253	0.480
Hidayah	38	31	14	32	61	25	107	59	125	111	0.910
Lee	103	78	17	89	65	16	284	112	243	97	0.718
Silva	96	125	36	159	218	80	317	197	536	378	0.718
Fernandez	27	42	20	29	54	18	96	82	112	90	0.711
Jafari	33	44	19	36	55	31	110	82	127	117	0.564
Varzari	60	142	65	61	128	52	262	272	250	232	0.613
Zhang	19	67	94	2	21	36	105	255	25	93	0.880

HWE, Hardy-Weinberg equilibrium.

### Test of heterogeneity

No or low heterogeneity was detected for the *Apa*I polymorphism, which included allele (a vs. A, *I* ^*2*^ = 23%, *p =* 0.25) homozygote (aa vs. AA, *I* ^*2*^ = 13%, *p* = 0.33), recessive (aa vs. Aa + AA, *I* ^*2*^ = 0%, *p* = 0.70), and dominant (aa + Aa vs. AA, *I* ^*2*^ = 30%, *p* = 0.19) genotype models. Similarly, the *Taq*I polymorphism exhibited low heterogeneity in the recessive model (tt vs. Tt + TT, *I* ^*2*^ = 33%, *p* = 0.12). Therefore, a fixed-effects model was applied to synthesize the *OR* for the *Apa*I polymorphism and a recessive model was used for the *Taq*I polymorphism. The remaining genotype models of *VDR* gene polymorphisms (i.e., *Fok*I, *Bsm*I, *Taq*I) were estimated to have substantial heterogeneity, indicating that a random-effects model could be applied to analyze the pooled *OR*.

### Quantitative synthesis

Table 2 presents the results of the analysis. For the *Fok*I *VDR* gene polymorphism, a significant association was observed with TB susceptibility in the allele model (f vs. F, *OR* = 1.235, 95%*CI*: 1.035–1.475, *p* = 0.019). A similar result was observed in the recessive model (ff vs. Ff + FF, *OR* = 1.317, 95%*CI*: 1.005–1.727, *p* = 0.046). However, no significant association with TB susceptibility was observed in the homozygote (ff vs. FF) and dominant (ff + Ff vs. FF) models. Regarding the other three VDR gene polymorphisms (BsmI, ApaI, TaqI), there was no evidence supporting a significant association between the four genotype models and TB susceptibility.

**FIGURE 3:**
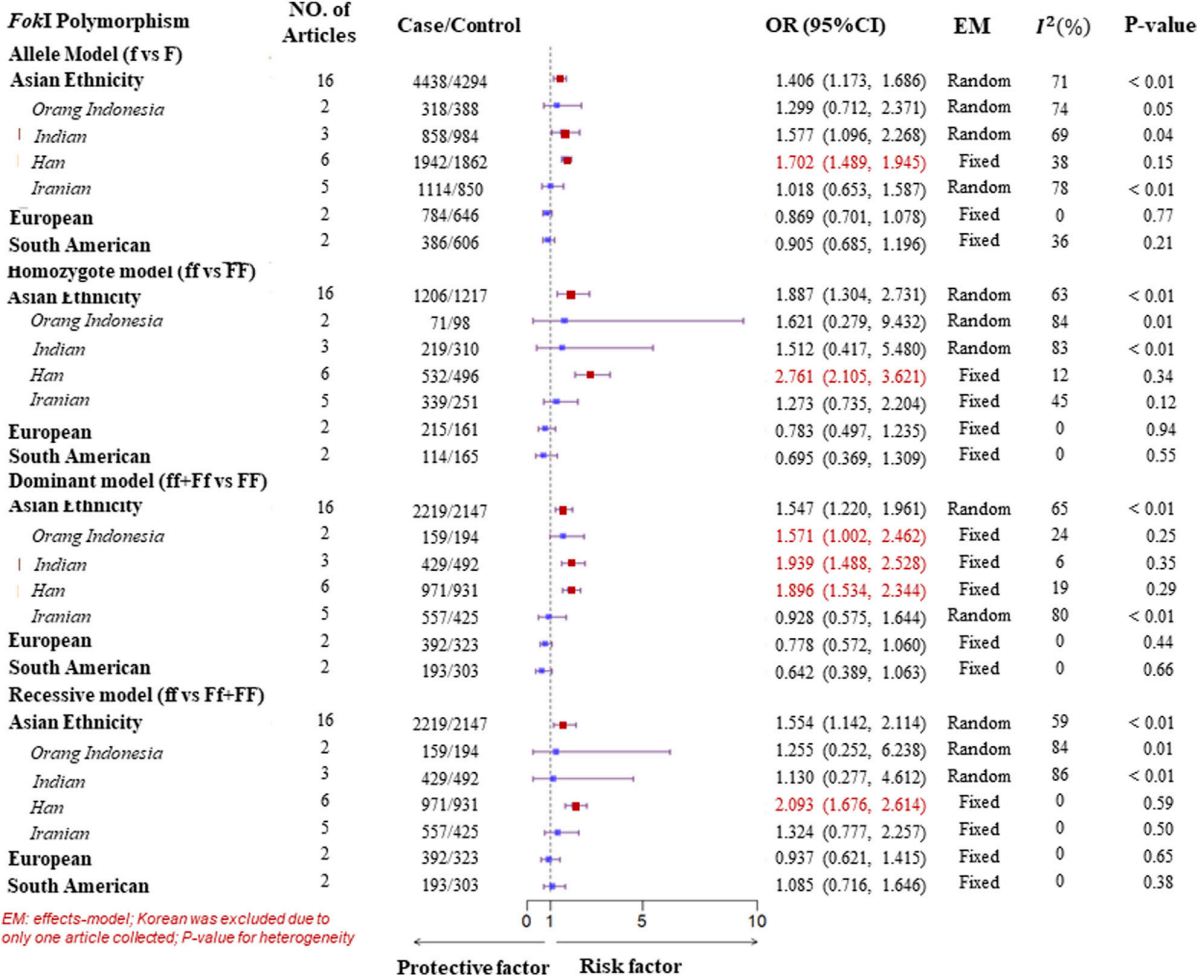
Subgroup analysis forest plot of four genotype models for the VDR gene *Fok*I polymorphism on ethnicity.

**FIGURE 4:**
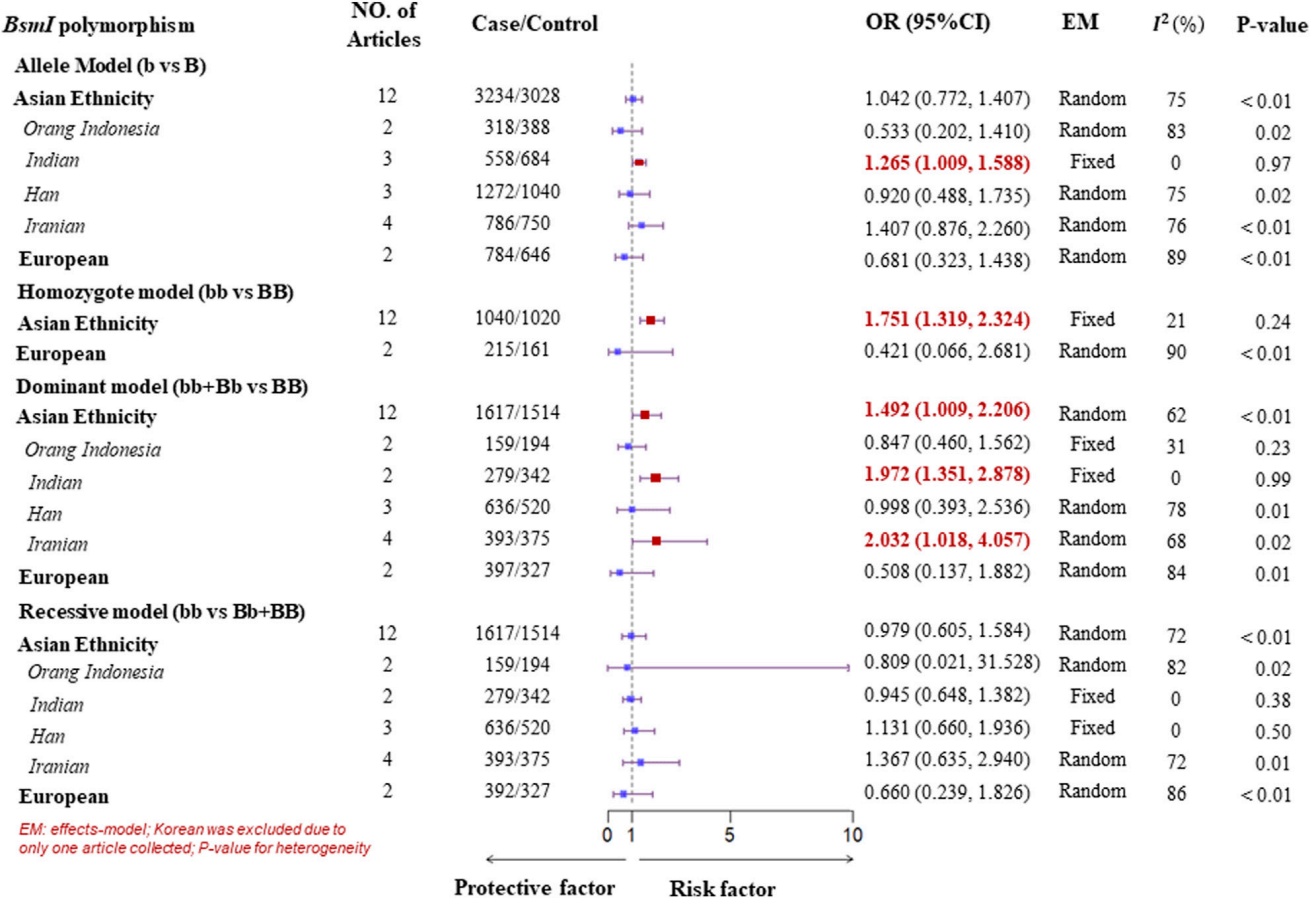
Subgroup analysis forest plot of four genotype models for the VDR gene *Bsm*I polymorphism on ethnicity.

A subgroup analysis of ethnicity was performed according to the four genotype models of the VDR gene polymorphisms. In the Asian population, for the FokI polymorphism, high heterogeneity was observed in all genotype models. Therefore, to detect more specific source of the heterogeneity, we classified Asian ethnicity as Orang Indonesia, Indian, Han population, and Iranian according to the district of the study populations. There was low or no heterogeneity in all genotype models for Han population. As a result, a Fixed-effects model was suggested to pool ORs, which showed a significant association of the FokI polymorphism with TB susceptibility in the Han population. Similarly, there was evidence of a significant association with TB susceptibility in the dominant model (ff + Ff vs. FF) for Orang Indonesia (OR = 1.571, 95%CI = 1.002–2.462, P_heterogeneity_ = 0.25, I^2^ = 24%) and Indian (OR = 1.939, 95%CI = 1.488–2.528, P_heterogeneity_ = 0.35, I^2^ = 6%). However, there was no significant association of the homozygote model and recessive model with TB susceptibility for Iranian although low heterogeneity was observed. Furthermore, evidence of a significant association with TB susceptibility was found within three genotype models of the *Bsm*I polymorphism in the Asian. One was a homozygote model (bb vs. BB) adopting a fixed-effects model to obtain a pooled *OR* = 1.751 (95%*CI*: 1.319–2.324) due to low heterogeneity (P_heterogeneity_ = 0.24, I^2^ = 21%). Another was the dominant model (bb + Bb vs. BB, P_heterogeneity_ < 0.01, I^2^ = 62%), in which it was a significant association with TB susceptibility in Indian (OR = 1.972, 95%CI = 1.351–2.878, generated from a fixed-effects model) with no heterogeneity (P_heterogeneity_ = 0.99, I^2^ = 0%). Besides, The result of the pooled ORs by a fixed-effects model displayed a significant association with TB in the allele model (b vs. B, OR = 1.265, 95%CI = 1.009–1.588, P_heterogeneity_ = 0.97, I^2^ = 0%) for Indian (Figures 3, 4). For the *Apa*I polymorphism, a significant association was found in the allele model (a vs. A, *OR* = 0.835, generated from a common-effects model, 95%*CI*: 0.712–0.979, P_heterogeneity_ < 0.32, I^2^ = 15%) and the homozygote model (aa vs. AA, *OR* = 0.702, generated from a common-effects model, 95%*CI* = 0.504–0.978, P_heterogeneity_ = 0.44, I^2^ = 0%) (Supplementary Figure S2).

However, no significant association between *VDR* gene polymorphisms and TB susceptibility was found in the South American or European populations (Figures 3, 4; Supplementary Figures S1–4). The details of the pooled *OR*s, heterogeneity tests, and Egger’s test for publication bias are shown in Table 3.

**TABLE 3:** The results of pooled ORs, test of heterogeneity and Egger’s test for publication bias in the four genotype models of VDR gene polymorphisms in the meta-analysis.

Polymorphism	No. of studies	Case/Control	Test of association			Test of heterogeneity		Egger’s test for publication bias	
			*OR*	95%*CI*	*p*-value	*I* ^2^ (%)	*p*-value	T	*p*-value
*Fok*I f vs. F	22	3052/3243	**1.235**	**(1.035–1.475)**	**0.019**	82.5	<0.001	0.98	0.337
ff + Ff vs. FF	22	3052/3243	1.275	(0.997–1.632)	0.053	79.0	<0.001	0.47	0.644
ff vs. Ff + FF	22	3052/3243	**1.317**	**(1.005–1.727)**	**0.046**	71.1	<0.001	0.31	0.756
ff vs. FF	22	3052/3243	1.427	(0.994–2.048)	0.054	79.2	<0.001	0.64	0.527
*Bsm*I b vs. B	15	2097/2209	0.976	(0.755–1.262)	0.853	77.2	<0.001	−1.04	0.317
bb + Bb vs. BB	15	2097/2209	1.233	(0.851–1.787)	0.269	70.3	<0.001	0.18	0.864
bb vs. Bb + BB	15	2097/2209	0.902	(0.613–1.326)	0.599	70.8	<0.001	0.76	0.463
bb vs. BB	15	2097/2209	1.364	(0.838–2.221)	0.212	59.3	0.002	0.39	0.704
*Taq*I t vs. T	15	2028/2047	1.005	(0.801–1.260)	0.968	62.3	0.001	−0.27	0.789
tt + Tt vs. TT	15	2028/2047	1.029	(0.821–1.299)	0.803	53.3	0.008	0.32	0.758
tt vs. Tt + TT	15	2028/2047	0.986	(0.742–1.310)	0.922	28.8	0.140	−0.13	0.897
tt vs. TT	15	2028/2047	1.085	(0.616–1.910)	0.778	50.4	0.013	0.04	0.970
*Apa*I a vs. A	8	1276/1506	0.913	(0.818–1.019)	0.106	23.0	0.246	NA	NA
aa + Aa vs. AA	8	1276/1506	0.891	(0.753–1.054)	0.178	30.1	0.187	NA	NA
aa vs. Aa + AA	8	1276/1506	0.885	(0.733–1.069)	0.206	00.0	0.705	NA	NA
aa vs. AA	8	1276/1506	0.836	(0.666–1.051)	0.125	13.1	0.328	NA	NA

The bold values indicate that the OR (95%CI) does not include 1, and the *p*-value is <0.05.

### Sensitivity analysis

Sensitivity analysis was performed to evaluate the impact of an individual article on the pooled *OR*s using the leave-one-out method, which involves omitting a single article each time. The results indicated an obvious decrease in the heterogeneity and significance of the pooled *OR* within the homozygote model of the *Bsm*I polymorphism after the deletion of one article (Jafari et al., 2016) (Supplementary Figure S5). Besides, no other individual article had a significant impact on the pooled *OR* or heterogeneity when omitted. This finding suggests that our results are relatively robust.

### Publication bias

**FIGURE 5:**
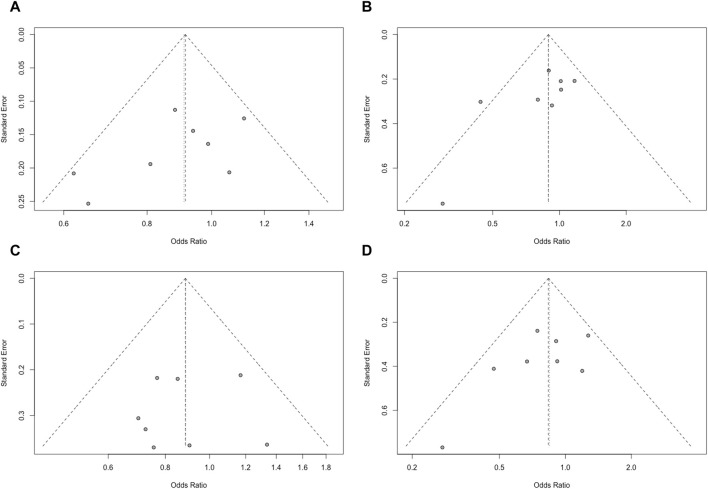
Funnel plot of the genotype models of the VDR gene *Apa*I polymorphism. [**(A)**: allele (a vs A); **(B)**: dominant (aa+Aa vs AA); **(C)**: recessive (aa vs Aa+AA); **(D)**: homozygote (aa vs AA)].

Publication bias was assessed using Egger’s test, which indicated no significant publication bias (*p* > 0.05) among in the included studies. Funnel plots were used to obtain the evidence of bias. No distinct asymmetry was found in the funnel plots, suggesting that there was no significant publication bias (Figure 5).

### Meta-regression

We finally performed a meta-regression analysis to explore the potential sources of heterogeneity among the *VDR* gene polymorphisms within the included articles. Our meta-regression analysis indicated that ethnicity could be a potential source of heterogeneity in the *Fok*I and *Bsm*I polymorphisms (i.e., within the homozygote and dominant models) of the VDR gene. However, the publication year was not the main source of heterogeneity. These details are presented in Table 4.

**TABLE 4:** The results of Meta-regression.

Heterogeneity source			*Coefficient*	95%*CI*	*Z*	*P*
*Fok*I (rs2228570)						
Ethnicity	Allele (f vs. F)	Asian (reference)	-	-	-	-
		European	−0.446	(-0.948,0.056)	−1.742	0.082
		North American	−0.876	(-1.523,-0.229)	−2.654	**0.008**
		South American	−0.408	(-0.934,0.118)	−1.521	0.128
	Dominant (ff + Ff vs. FF)	Asian (reference)	-	-	-	-
		European	−0.615	(-1.234,0.003)	−1.951	0.051
		North American	−1.236	(-2.041,-0.431)	−3.010	**0.003**
		South American	−0.834	(-1.569, −0.098)	−2.221	**0.026**
	Recessive (ff vs. Ff + FF)	Asian (reference)	-	-	-	-
		European	−0.553	(-1.400,0.295)	−1.278	0.201
		North American	−1.011	(-2.021, −0.001)	−1.961	**0.049**
		South American	−0.390	(-1.230,0.450)	−0.911	0.363
	Homozygote (ff vs. FF)	Asian (reference)	-	-	-	-
		European	−0.875	(-1.875,0.124)	−1.716	0.086
		North American	−1.619	(-2.857, −0.381)	−2.563	**0.010**
		South American	−0.955	(-2.020,0.110)	−1.758	0.079
Publication year		f vs. F	0.011	(-0.0257,0.0485)	0.602	0.548
		ff + Ff vs. FF	0.021	(-0.032,0.073)	0.761	0.447
		ff vs. Ff + FF	0.001	(-0.053,0.056)	0.052	0.959
		ff vs. FF	0.012	(−0.064,0.087)	0.306	0.760
*Bsm*I (rs1544410)						
Ethnicity	Allele (b vs. B)	Asian (reference)	-	-	-	-
		European	−0.480	(-1.275,0.315)	−1.183	0.237
		North American	0.012	(-1.033,1.057)	0.023	0.982
	Dominant (bb + Bb vs. BB)	Asian (reference)	-	-	-	-
		European	−1.075	(-2.105, −0.046)	−2.048	**0.041**
		North American	−0.320	(-1.566,0.926)	−0.504	0.614
	Recessive (bb vs. Bb + BB)	Asian (reference)	-	-	-	-
Heterogeneity source			*Coefficient*	95%*CI*	*Z*	*P*
	Homozygote (bb vs. BB)	European	−0.452	(-1.681,0.777)	−0.720	0.472
		North American	−0.048	(-1.833,1.736)	−0.053	0.958
		Asian (reference)	-	-	-	-
		European	−1.407	(-2.598, −0.217)	−2.317	**0.021**
		North American	−0.690	(-2.323,0.943)	−0.828	0.408
Publication year		b vs. B	−0.010	(-0.082,0.063)	−0.268	0.789
		bb + Bb vs. BB	−0.061	(-0.158,0.037)	−1.223	0.221
		bb vs. Bb + BB	0.023	(-0.087,0.134)	0.409	0.683
		bb vs. BB	−0.021	(-0.158,0.117)	−0.293	0.770
*Taq*I (rs731236)						
Ethnicity	Allele (t vs. T)	Asian (reference)	-	-	-	-
		European	−0.456	(-1.499,0.588)	−0.856	0.392
		North American	−0.003	(-0.887,0.881)	−0.007	0.994
		South American	−0.332	(-1.103,0.439)	−0.844	0.399
	Dominant (tt + Tt vs. TT)	Asian (reference)	-	-	-	-
		European	−0.468	(-1.712,0.776)	−0.738	0.461
		North American	−0.059	(-1.074,0.956)	−0.113	0.910
		South American	−0.431	(-1.304,0.443)	−0.966	0.334
	Recessive (tt vs. Tt + TT)	Asian (reference)	-	-	-	-
		European	−1.283	(-2.917,0.351)	−1.539	0.124
		North American	0.085	(-1.118,1.287)	0.138	0.890
		South American	0.115	(-1.988,2.218)	0.107	0.915
	Homozygote (tt vs. TT)	Asian (reference)	-	-	-	-
		European	−1.715	(-3.690,0.261)	−1.701	0.089
		North American	−0.001	(-1.502,1.500)	−0.001	0.999
		South American	−0.148	(-2.367,2.071)	−0.131	0.896
Publication year		t vs. T	−0.013	(-0.058,0.033)	−0.549	0.583
		tt + Tt vs. TT	−0.005	(-0.052,0.042)	−0.214	0.830
		tt vs. Tt + TT	−0.086	(-0.184,0.012)	−1.728	0.084
		tt vs. TT	−0.097	(-0.215,0.021)	−1.612	0.107

The bold values indicate that the OR (95%CI) does not include 1, and the *p*-value is <0.05.

## Discussion

The findings presented in this paper could provide clues for preventing TB from the perspective of vitamin D supplementation, which is a controversial topic in the field of medicine and health. In this meta-analysis, we pooled the results of 25 published articles to assess the association between various genotype models of VDR gene polymorphisms and TB susceptibility. We found that there was a significant association between an increased risk of developing TB and the allele (f vs. F) and recessive (ff vs. Ff + FF) models of the FokI polymorphism, whereas there was no evidence that the homozygote (ff vs. FF) and dominant (ff + Ff vs. FF) models were associated with TB risk. Further analysis based on Asian ethnicity revealed a significant association, in which all genotype models of the VDR FokI polymorphism contributed to the risk of developing TB in the Han population. It was observed there has likewise correlation for Orang Indonesia and Indian in the dominant model (ff + Ff vs. FF). However, a significant association between the ApaI polymorphism in VDR and a reduced risk of TB was found in the allele model (a vs. A) and the homozygote model (aa vs. AA). A possible reason for these inconsistent findings is that individuals are exposed to different environmental factors that could affect their genetic susceptibility to TB. However, further relevant studies are required to support this viewpoint.

Previous meta-analyses have evaluated the role of VDR gene polymorphisms in TB risk. Regarding the FokI polymorphism, some meta-analyses (Xu and Shen, 2019; Mohammadi et al., 2020) found no significant association between the FokI polymorphism and TB susceptibility. However, Cao et al. (2016) and Yadav et al. (2021) found evidence of an association in the homozygote (ff vs. Ff) and recessive (ff vs. Ff + FF) models. In addition, two meta-analyses (Chen et al., 2013; Huang et al., 2015) merely found that the f allele might contribute to the risk of TB in a recessive model (ff vs. Ff + FF), and our findings were consistent with this result. The FokI polymorphism, located in exon 2 at the translation initiation site of the VDR gene, produces two different receptor proteins. The F allele, linked to the expression of a shorter protein of 424 amino acids, displays higher transcriptional activity than another protein of 427 amino acids encoded by the f allele (Ruiz-Ballesteros et al., 2020). Therefore, the f allele of FokI could potentially decrease the activity of the VDR protein, thereby obstructing the interaction between active vitamin D and VDR, which might ultimately contribute to susceptibility to TB.

With respect to the BsmI polymorphism, no significant association was observed in this study. However, we found evidence to support an increased risk of TB in the homozygote model (bb vs. BB, OR = 1.751, 95%CI: 1.319–2.324) and the dominant model (bb + Bb vs. BB, OR = 1.492, 95%CI: 1.009–2.206) in Asian (Figure 4). More specific findings of Han population and Indian showed a significant association of the dominant model with the risk of TB. A similar meta-analysis performed by Wu et al. (2013a) demonstrated a significant association between the VDR gene BsmI polymorphism and a decreased TB risk within all four genotype models, and a similar association was found in Asians. One possible reason for the inconsistency in these findings is the lack of strict inclusion and exclusion criteria. For example, this previous study did not provide the criteria for excluding HIV-positive populations, as individuals with TB can be co-infected with this virus. Finally, the accuracy of a TB diagnosis is reduced in the HIV-positive population (Bell and Noursadeghi, 2018). Furthermore, a relevant assessment of the literature quality for case-control studies was not found in any previous study. Hence, low-quality articles could have generated biased results and might have further affected the pooled effects of the meta-analysis. Another reason could be the statistical power, which normally deviates with sample sizes; therefore, more relevant studies should be conducted in the future to examine our inconsistent results.

Regarding the association between the TaqI polymorphism in the VDR gene and the risk of TB, this association was not found within any of the four genotype models of the TaqI polymorphism Möller and Hoal, 2010. This is consistent with the results of a meta-analysis by Areeshi et al (Areeshi et al., 2017). A possible explanation for this is that the t allele of the VDR TaqI polymorphism is likely involved in the active disease process, whereas the variant does not act as a primary polymorphism with respect to TB infection. However, the TaqI polymorphism in the VDR gene was found to play a role in TB development in another meta-analysis performed by Xu and Shen (2019) in 2019; however, this meta-analysis did not exclude the HIV-positive population, which could have generated bias in terms of the pooled effect.

This meta-analysis had several strengths compared to previous studies. First, we performed the meta-analysis using relatively rigorous inclusion and exclusion criteria. Therefore, only high-quality articles including HIV-negative populations and those adhering to the HWE for gene distribution were eligible for the analysis. In other words, we avoided confounding factors that might have biased the pooled effect. Another strength is that we performed a meta-regression analysis of potential sources of heterogeneity among articles. Moreover, we anticipate performing more relevant analyses to explore other possible heterogeneity sources, such as the sample size or type of TB. However, our study has some limitations that should be acknowledged. First, despite the rigorous inclusion and exclusion criteria adhered to in this meta-analysis, our sample size was relatively small. Consequently, more studies with similar criteria are required to validate our pooled results. Second, in the present meta-analysis, we included articles published in English only, from three electronic databases (PubMed, Embase, and Medline). This could introduce a potential bias if studies in other languages or those indexed in other databases are missed. It should be noted that the impact of gene–environment interactions on the susceptibility to TB was also not considered in the present study. Furthermore, we anticipate performing Genome-wide association studies (GWAS) to identify a robust correlation between VDR gene polymorphisms and TB susceptibility in future research.

## Conclusion

In summary, this meta-analysis adhered to strict inclusion and exclusion criteria to systematically evaluate the association between *VDR* gene polymorphisms and TB risk in the HIV-negative population. The *Fok*I polymorphism was found to be associated with an increased risk of TB in the overall analysis. This indicates that the f allele could contribute more to TB risk than the F allele, particularly in Asians. However, the *Apa*I polymorphism was determined to play a protective role against TB. Further large-scale studies are required to classify the role of ethnicity and other potential factors in the relationship between *VDR* gene polymorphisms and TB susceptibility.

## Data Availability

The original contributions presented in the study are included in the article/Supplementary Material, further inquiries can be directed to the corresponding authors.
