# Supplementary material for: Association between vitamin D receptor gene polymorphisms and susceptibility to tuberculosis: a systematic review and meta-analysis
Source: Front Genet. 2024 Aug 20;15:1382957. doi: 10.3389/fgene.2024.1382957 (PMC11368754; doi:10.3389/fgene.2024.1382957)
Supplement: Supplementary file 1 [file Table1.DOCX]

**Supplementary Materials：**

1. Search strategy：

| Literature Search Strategies (PubMed) | | |
| --- | --- | --- |
| Step code | Search terms | Result |
| Tuberculosis |  |  |
| #1 | "Tuberculosis"[Majr] | 183,945 |
| #2 | (((((((Tuberculoses) OR (Kochs Disease)) OR (Koch's Disease)) OR (Koch Disease)) OR (Mycobacterium tuberculosis Infection)) OR (Infection, Mycobacterium tuberculosis)) OR (Infections, Mycobacterium tuberculosis)) OR (Mycobacterium tuberculosis Infections) | 300,938 |
| #3 | #1 OR #2 | 300,938 |
| Genetic polymorphism |  |  |
| #4 | "Polymorphism, Genetic"[Majr] | 134,906 |
| #5 | ((((((((Polymorphisms, Genetic) AND (Genetic Polymorphism)) AND (Genetic Polymorphisms)) AND (Gene Polymorphism)) AND (Gene Polymorphisms)) AND (Polymorphism, Gene)) AND (Polymorphisms, Gene)) AND (Polymorphism (Genetics))) AND (Polymorphisms (Genetics)) | 331,539 |
| #6 | #4 OR #5 | 411,596 |
| #7 | (Vitamin D Receptor) OR (VDR) | 13,964 |
| Excluding |  |  |
| #8 | "Editorial" [Publication Type] | 650,146 |
| #9 | "Letter" [Publication Type] | 1,217,468 |
| #10 | (#3 AND #6 AND #7) NOT #8 NOT #9 | 154 |

| Literature Search Strategies (Embase) | | |
| --- | --- | --- |
| **Step code** | **Search terms** | **Result** |
| **Tuberculosis** |  |  |
| **#1** | **'tuberculosis'/exp OR tuberculosis** | **390,895** |
| **#2** | **tuberculosis:ti,ab,kw** | **262,106** |
| **#3** | **tubercul*:ti,ab,kw** | **303,381** |
| **Genetic polymorphism** |  |  |
| **#4** | **'genetic polymorphism'/exp OR 'genetic polymorphism' OR (('genetic'/exp OR genetic) AND ('polymorphism'/exp OR polymorphism))** | **528,780** |
| **#5** | **'genetic polymorphism':ti,ab,kw** | **18,049** |
| **#6** | **'gene* polymorph*':ti,ab,kw** | **87,527** |
| **#7** | **'vitamin d receptor'/exp OR 'vitamin d receptor' OR 'vdr'** | **19,628** |
| **#8** | **'vitamin d receptor':ti,ab,kw** | **12,146** |
| **#9** | **'vdr':ti,ab,kw** | **11,601** |
| **#10** | **(#1 OR #2 OR #3) AND (#4 OR #5 OR #6) AND (#7 OR #8 OR #9)** | **268** |
| **Excluding** |  |  |
| **#11** | **editorial: ti,ab,kw** | **116,992** |
| **#12** | **'editorial'/exp OR 'editorial'** | **982,737** |
| **#13** | **letter:ti,ab,kw** | **117,380** |
| **#14** | **'letter'/exp OR 'letter'** | **1,365,508** |
| **#15** | **#11 OR #12** | **982,737** |
| **#16** | **#13 OR #14** | **1,365,508** |
| **#17** | **#10 NOT #15 NOT #16** | **253** |

| Literature Search Strategies (Medline) | | |
| --- | --- | --- |
| Step code | Search terms | Result |
| Tuberculosis |  |  |
| #1 | exp tuberculosis/ or tuberculosis.mp. | 278,845 |
| #2 | "tubercul*".ab,ti,kw. | 262,622 |
| Genetic polymorphism |  |  |
| #3 | genetic polymorphism.mp. or exp polymorphism,Genetic/ | 305810 |
| #4 | "gen* polymorph*".ab,ti,kw. | 63116 |
| #5 | **exp vitamin D receptor/ or vitamin D receptor.mp.** | 11587 |
| #6 | "VDR".ab,ti,kw. | 8311 |
| #7 | (#1 or #2) and (#3 or #4) and (#5 or #6) | 139 |
| Excluding |  |  |
| #8 | (letter or meeting abstract).pt. | 1,218,003 |
| #9 | #7 not #8 | 136 |

2. Subgroup analysis forest plots


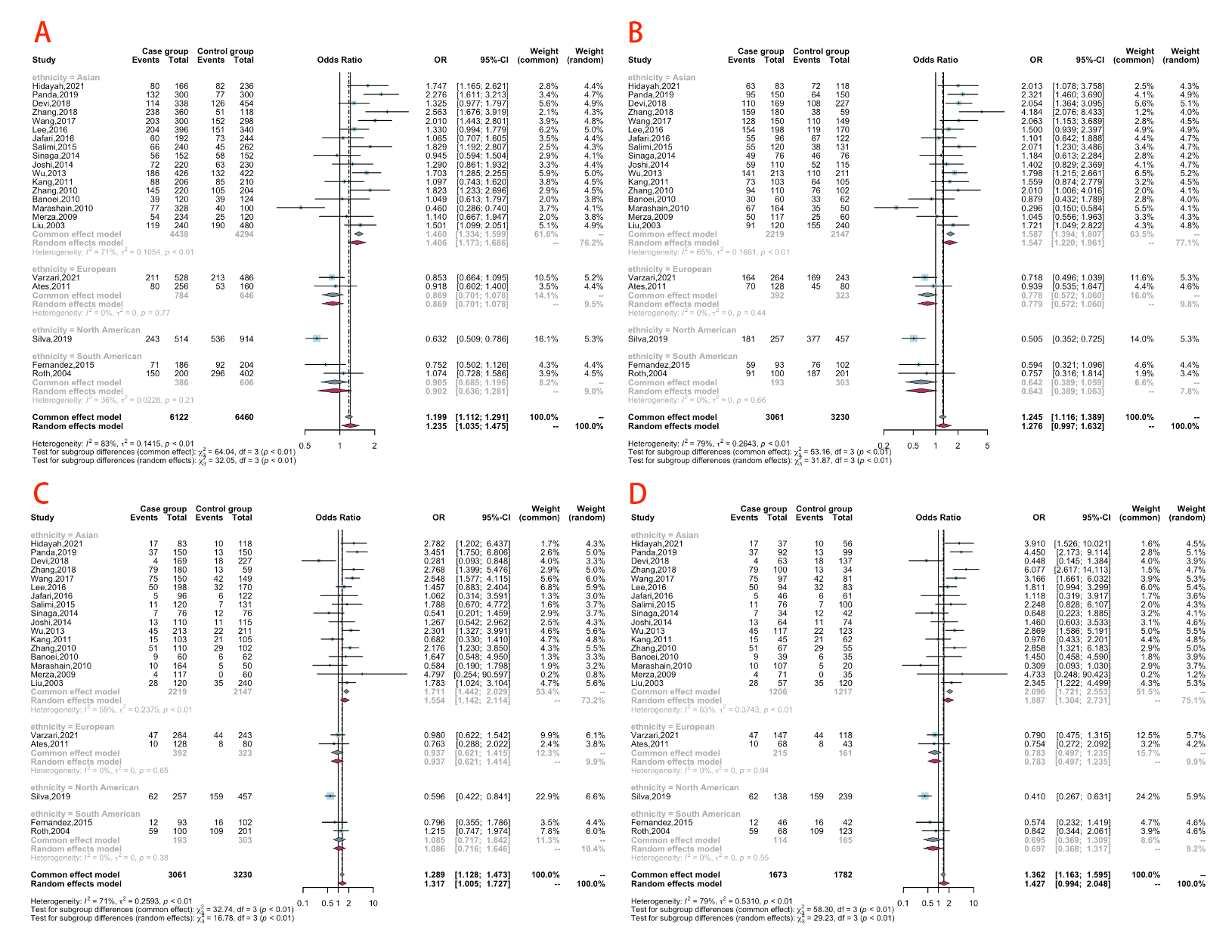


Figure S1. Subgroup analysis forest plot of four genotype models for the *VDR* gene *Fok*I polymorphism on ethnicity within included articles. [A: allele: f vs F; B: dominant (ff+Ff vs FF); C: recessive (ff vs Ff+FF); D: homozygote (ff vs FF)]


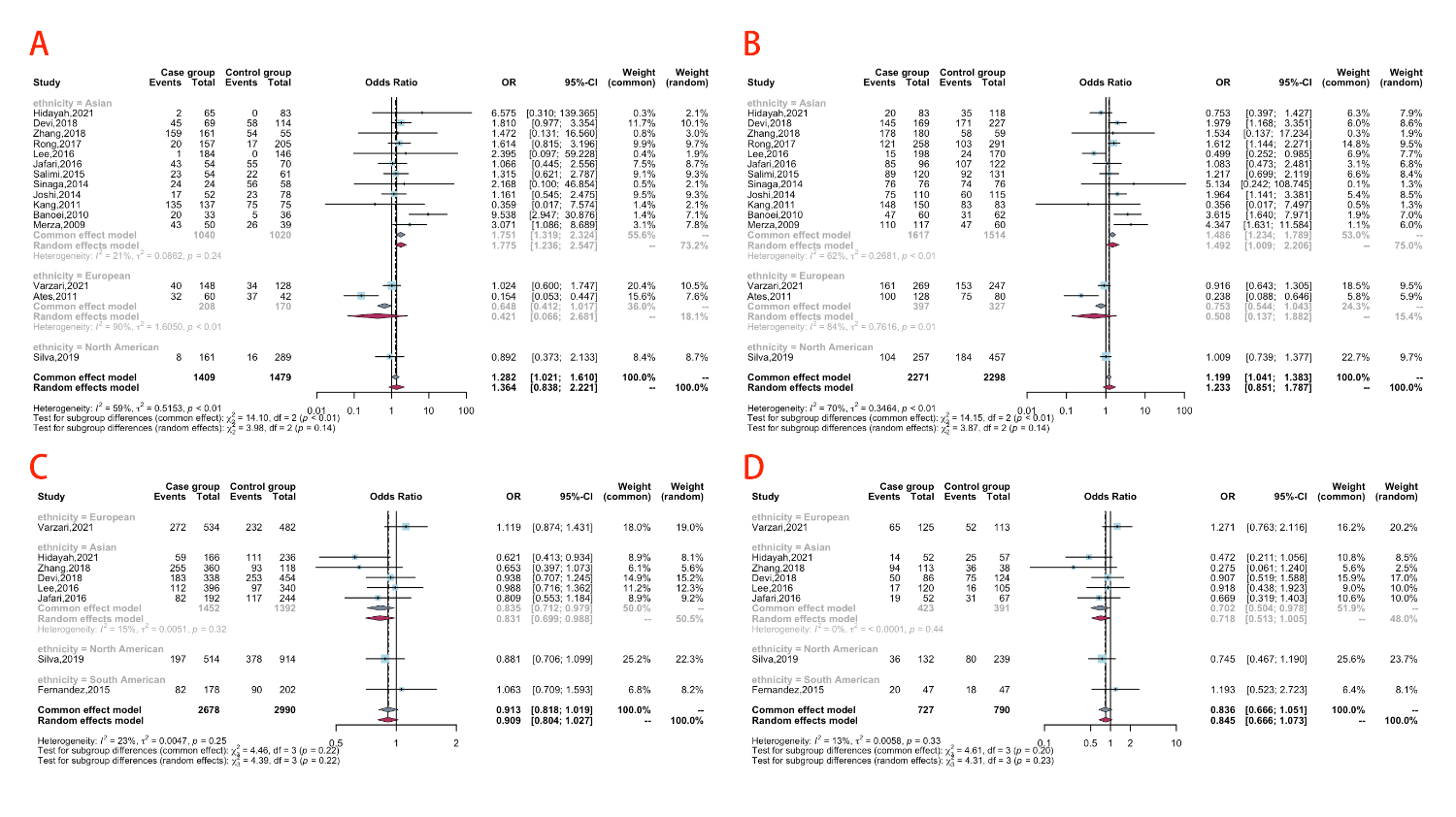


Figure S2. Subgroup analysis forest plot of significant genotype models for the *VDR* gene *Bsm*I and *Apa*I polymorphisms on ethnicity within included articles. [A: *Bsm*I homozygote: bb vs BB; B: *Bsm*I dominant (bb+Bb vs BB); C: *Apa*I allele (a vs A); D: *Apa*I homozygote (aa vs AA)]


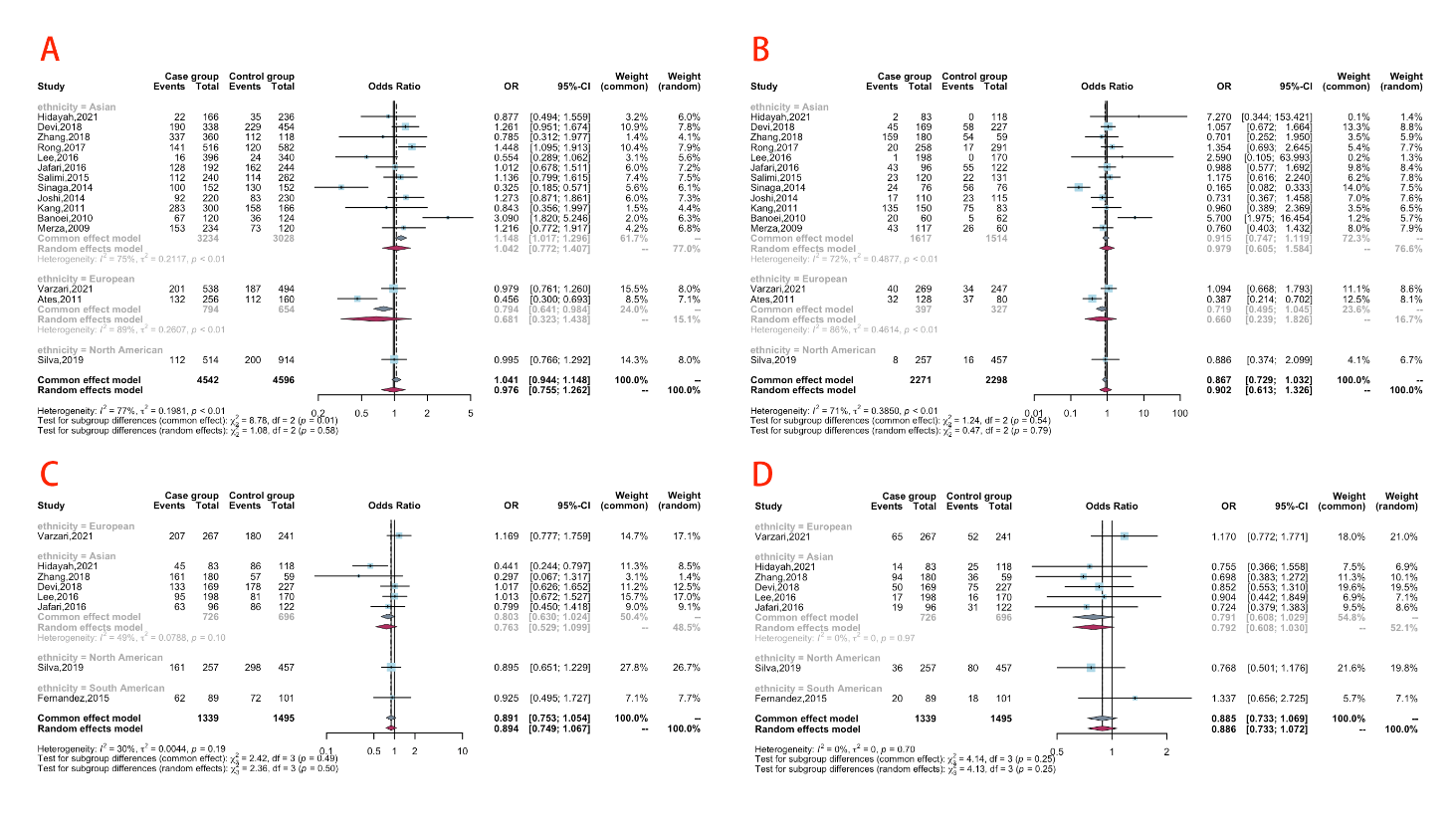


Figure S3. Subgroup analysis forest plot of other genotype models for the *VDR* gene *Bsm*I and *Apa*I polymorphisms on ethnicity within included articles. [A: *Bsm*I allele: b vs B; B: *Bsm*I recessive (bb vs Bb+BB); C: *Apa*I dominant (aa+Aa vs AA); D: *Apa*I recessive (aa vs Aa+AA)]

**
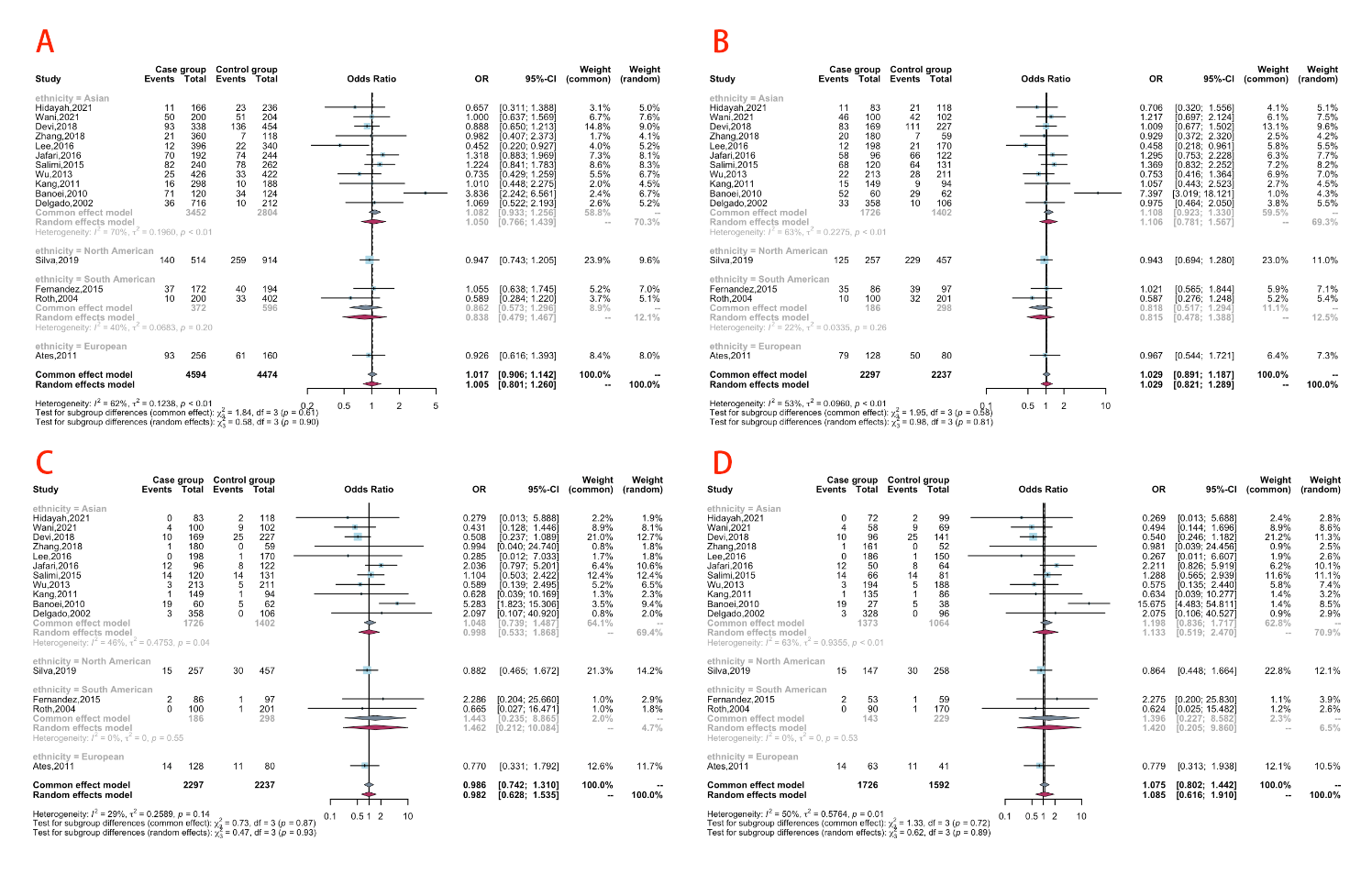
**

Figure S4. Subgroup analysis forest plot of four genotype models of the *VDR* gene *Taq*I polymorphism on ethnicity within included articles. [A: allele (t vs T); B: dominant (tt+Tt vs TT); C: recessive (tt vs Tt+TT); D: homozygote (tt vs TT)]


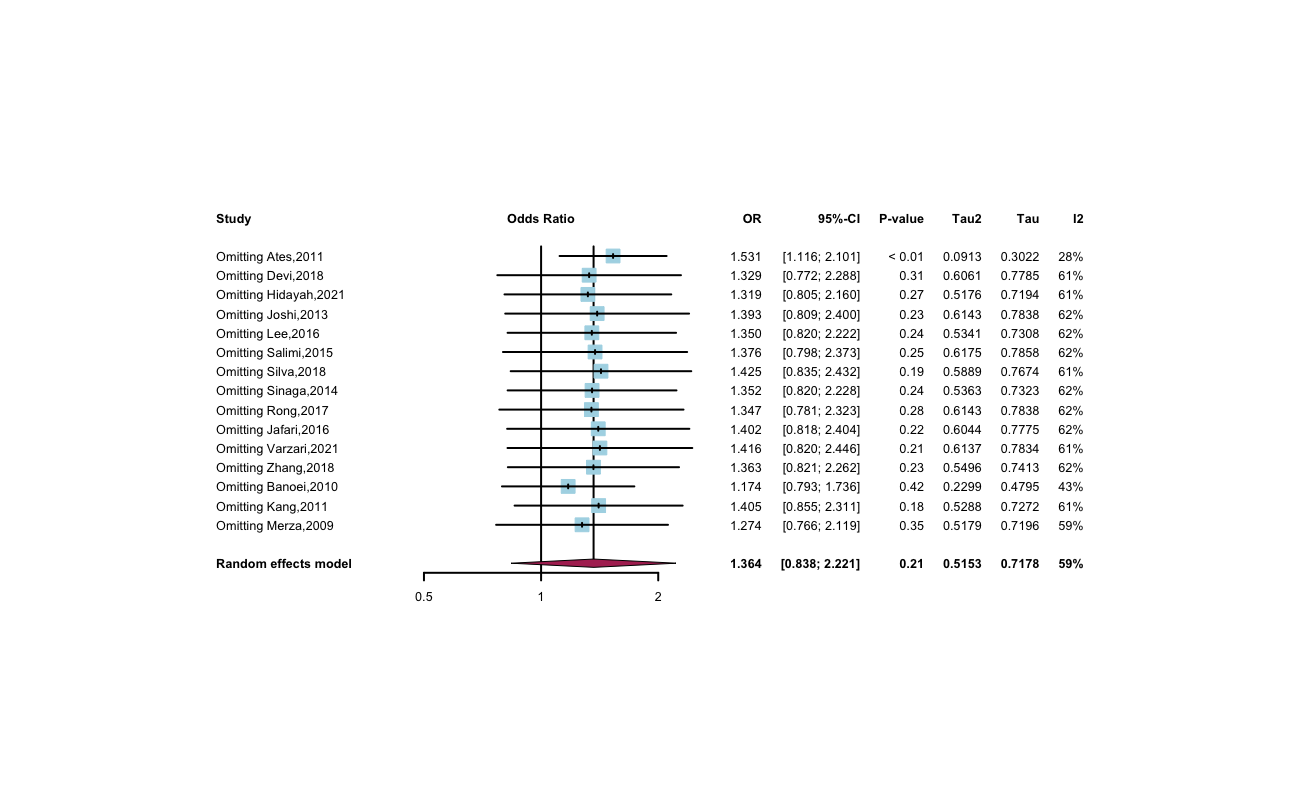


Figure S5. Sensitivity analysis forest plot of the homozygote model of the *VDR* gene *Bsm*I polymorphism.
